# Management of acute variceal hemorrhage

**DOI:** 10.12688/f1000research.18807.1

**Published:** 2019-06-25

**Authors:** Alberto Zanetto, Guadalupe Garcia-Tsao

**Affiliations:** 1 Digestive Disease Section, Yale University School of Medicine, 333 Cedar Street 1080 LMP New Haven, Connecticut, 06520-8019, USA; 2 VA-CT Healthcare System, 950 Campbell Ave, West Haven, Connecticut, 06516, USA

**Keywords:** cirrhosis, portal hypertension, variceal bleeding, TIPS, non-selective betablockers

## Abstract

Gastrointestinal bleeding is one of the major causes of death in patients with cirrhosis, and gastroesophageal varices represent the main source of hemorrhage. Even though in the last decades survival has been improved because of the widespread adoption of effective treatments and optimization of general medical care, mortality is still significantly high, and decompensated patients pose a complex challenge requiring a multidisciplinary approach that is crucial to improve survival. The aims of this commentary are to review the most recent advances in the management of esophageal variceal bleeding and to highlight useful information to aid hepatologists in clinical practice.

## Introduction

Gastrointestinal (GI) hemorrhage constitutes the second most frequent decompensating event in patients with cirrhosis ^1, 2^, and the main source is esophageal varices. Despite advances in its management, esophageal variceal hemorrhage (VH) is still associated with a 6-week mortality rate of 10 to 20% ^1– 3^. This review focuses on the management of patients presenting with esophageal VH, including both the treatment of the acute event and strategies to prevent recurrent bleeding.

## Control of hemorrhage

The main aims of therapy in a hospitalized patient with cirrhosis with acute upper GI hemorrhage are to control hemorrhage and to prevent early re-bleeding and death. Management can be divided into general measures (before the source of hemorrhage has been determined) and measures that are specific once endoscopy has determined that hemorrhage is from esophageal varices.

### General management of upper gastrointestinal hemorrhage

In addition to protecting the circulatory and respiratory status and starting proton pump inhibitors (PPIs), as recommended in any patient with upper GI hemorrhage, nuances in the general management of patients with cirrhosis include a cautious transfusion strategy and use of prophylactic antibiotics. Other measures that will be discussed are the need to correct coagulopathy and the use of PPIs in cirrhosis.

***Blood transfusion strategy.*** Patients with cirrhosis have a hyperdynamic circulatory state. In a way, loss of intravascular volume through hemorrhage reduces portal pressure, leading to cessation of active hemorrhage. Restitution of intravascular volume can induce a rebound increase in portal pressure, which may lead to failure to control bleeding or re-bleeding or both ^4, 5^. In fact, in a landmark randomized control trial (RCT) ^6^, a “restrictive” transfusion strategy—hemoglobin (Hb) threshold for transfusion of 7 g/dL with a target range of 7 to 9 g/dL—was associated with a higher survival than a “liberal” strategy (Hb threshold for transfusion of 9 g/dL with a target range of 9 to 11 g/dL). This effect on survival was significant in Child A and B patients but not in those belonging to the Child C class, although a clear trend toward a decrease in re-bleeding was demonstrated in all patients with cirrhosis. Therefore, current guidelines recommend initiating transfusions when Hb levels decrease to less than 7 g/dL, and the target level is 7 to 9 g/dL ^3, 7, 8^.

***Antibiotic prophylaxis.*** Bacterial infections are reported in more than 50% of patients with cirrhosis and GI bleeding and are associated with failure to control bleeding, high risk of re-bleeding, and increased mortality ^9– 12^. Therefore, timely short-term antibiotic prophylaxis is an essential step in the management of these patients ^7, 8^. Prophylaxis must be instituted as early as VH is suspected, and timely administration has been associated with a reduced re-bleeding rate and lower mortality ^13^. The importance of prophylaxis is incontrovertible in patients with advanced cirrhosis, whereas in patients with less severe disease, conflicting data have been published. In a retrospective study, Child A patients had a low rate of bacterial infection (2%) in the absence of antibiotic prophylaxis, and there was no difference in mortality between patients on and off antibiotics ^14^. In contrast, antibiotics were associated with a marked mortality reduction in Child C patients ^14^. However, more prospective studies are needed to assess whether antibiotic prophylaxis can be avoided in Child A patients ^3^.

Intravenous (IV) ceftriaxone (1 g/24 hours) for a maximum of 7 days is the first choice in patients with advanced cirrhosis, in patients receiving quinolone prophylaxis, and in hospitals where there is a high frequency of quinolone-resistant bacteria strains. Norfloxacin 400 mg twice a day may be used in the other patients and is rationally sound since it achieves selective intestinal decontamination. However, norfloxacin is no longer available in the US or in most in-patient formularies. Because of this and because quinolone resistance is widespread, the third-generation cephalosporin ceftriaxone is the prophylactic antibiotic of choice ^7, 8^. Local microbial epidemiology and resistance patterns may optimize the choice of antibiotic prophylaxis in specific centers. Prophylactic antibiotics should be used for a maximum of 7 days (consider discontinuing when hemorrhage has resolved and vasoactive drugs discontinued) ^8^ and their use should not be extended after discharge from the hospital.

***Other measures***

**Correction of coagulopathy** In patients with cirrhosis, particularly at a decompensated stage, prothrombin time (PT) does not reflect bleeding tendency ^15^, and correction of international normalized ratio with fresh frozen plasma should not be performed in case of VH. In fact, the administration of recombinant activated factor VII, which can revert PT prolongation, did not show an additional beneficial effect to standard therapy in two RCTs ^16, 17^ and is not recommended. No specific data are available regarding the management of thrombocytopenia in the setting of VH and therefore no recommendation can be made. Desmopressin increases levels of factor VIII and von Willebrand factor and its administration shortened bleeding time in a small study of compensated patients ^18^. In an RCT, no differences in control of VH were found between patients randomly assigned to terlipressin alone versus those who received terlipressin plus desmopressin ^19^. Therefore, desmopressin is not currently recommended.

**Proton pump inhibitors** IV PPIs should be initiated in case of upper GI hemorrhage because peptic ulcers are the cause of bleeding in one third of the cases ^20^. However, when portal hypertensive bleeding is confirmed, PPIs should be discontinued. Limited evidence suggests that short-term use (10 days) of PPIs might reduce banding ulcer size ^21^ without having a significant effect on bleeding. Because PPIs are associated with an increased risk of hepatic encephalopathy ^22^, especially in those with recent bacterial infections ^23^ and with a high risk of 30-day re-admission ^24^, their use (if at all) should not be extended past the hospitalization period.

### Specific management of acute esophageal variceal hemorrhage

Standard therapy for VH involves the use of IV splanchnic vasoconstrictors, which will decrease portal pressure acutely by decreasing flow into the portal venous system, and the placement of rubber bands around esophageal varices, particularly the varix that is assumed to be the source, in order to physically contain the hemorrhage. The transjugular intrahepatic portosystemic shunt (TIPS), which connects the hypertensive portal vein to a normotensive systemic vein (the inferior vena cava), will quickly normalize pressure in the main portal vein and is used in patients who fail standard therapy either because bleeding cannot be controlled or because of recurrence of bleeding (rescue TIPS) or in those at a high risk of failing standard therapy (pre-emptive TIPS).

***Intravenous splanchnic vasoconstrictors.*** Three IV vasoconstrictors are recommended: terlipressin, somatostatin, or octreotide ^7, 8^. In a 2012 meta-analysis ^25^, use of IV vasoconstrictors was associated with a higher probability of bleeding control and with a lower 7-day mortality rate than in untreated patients. An IV vasoconstrictor must be initiated as soon as early administration is associated with improved survival ^26^. Availability and cost guide the choice of which of the three vasoconstrictors to use in clinical practice. In the US, octreotide is the only available drug. Vasoconstrictors should be continued up to 5 days after the confirmation of VH ^7, 8^. The feasibility of a shorter administration (that is, 24 to 48 hours versus 3 to 5 days) was evaluated in a recent meta-analysis ^27^. Although the risk of 42-day mortality was not significantly different, risk stratification was lacking. Indeed, it may be the case that patients with less advanced disease (Child A) can receive a shorter duration of therapy but that all others require 5 days, but further studies are needed to answer this question. Therefore, guidelines recommend that IV vasoconstrictors must be initiated as soon as possible (prior to diagnostic endoscopy) and be administered for 2 to 5 days ^3, 7, 8^.

***Endoscopic variceal ligation.*** Once hemodynamic stability has been reached, upper endoscopy must be performed to determine the cause of bleeding and to provide specific endoscopic treatment ^3, 7, 8^. Timing is important, and delayed endoscopy (that is, more than 15 hours) has been correlated with increased risk of death ^28^. Combined treatment with endoscopic variceal ligation (EVL) and IV vasoconstrictors is recommended as the standard of care ^3, 7, 8^. Sclerotherapy may be considered in rare cases in which EVL is not technically feasible. Cyanoacrylate glue injection is not recommended in the control of esophageal VH but plays an important role in the control of gastric fundal VH ^7^. Hemostatic powder applied endoscopically may be useful in the control of hemorrhage but EVL would still be needed and therefore its applicability remains to be determined ^29^.

In patients with uncontrolled bleeding, guidelines recommend placement of balloon tamponade. However, it carries a high risk of complications and must be considered only a temporary (maximum of 24 hours) bridge to definitive treatment (for example, TIPS) ^3^. It was recently shown that placement of a self-expandable esophageal metal stent (placed orally or endoscopically) was associated with greater bleeding control and lower adverse events compared with balloon tamponade ^30^ and therefore should be preferred in sites where it is available. Because these stents may remain in place for up to 7 days, placement allows more time to plan for a definitive therapy. These stents are not approved by the US Food and Drug Administration for use in the US.

Guidelines recommend that endoscopy be performed as early as possible once hemodynamic stability has been achieved and not more than 12 hours after presentation ^3, 8^. When VH is confirmed, by the presence of a spurting varix, a clot, or a “white nipple” overlying the varix or when varices are the only abnormality observed that would explain the bleed, all esophageal varices should be ligated, particularly the one that is considered the source of hemorrhage.

***TIPS***

**Rescue TIPS in patients who fail standard therapy** Notwithstanding therapy with antibiotics, vasoconstrictors, and EVL, 10 to 15% of patients will present persistent bleeding or early re-bleeding that is associated with high risk of death ^31^. Negative and independent predictive factors include a hepatic venous pressure gradient (HVPG) of more than 20 mm Hg, Child C class, portal vein thrombosis (PVT), and systolic blood pressure of less than 100 mm Hg at admission ^32, 33^. Rescue TIPS is the first-line therapy in patients who have persistent severe bleeding or early variceal re-bleeding ^3, 8^, and patient selection is relevant. Indeed, in patients with very advanced liver disease, rescue TIPS may be futile in patients with a Child–Pugh score of 14 to 15 ^34^.

**Figure 1.**
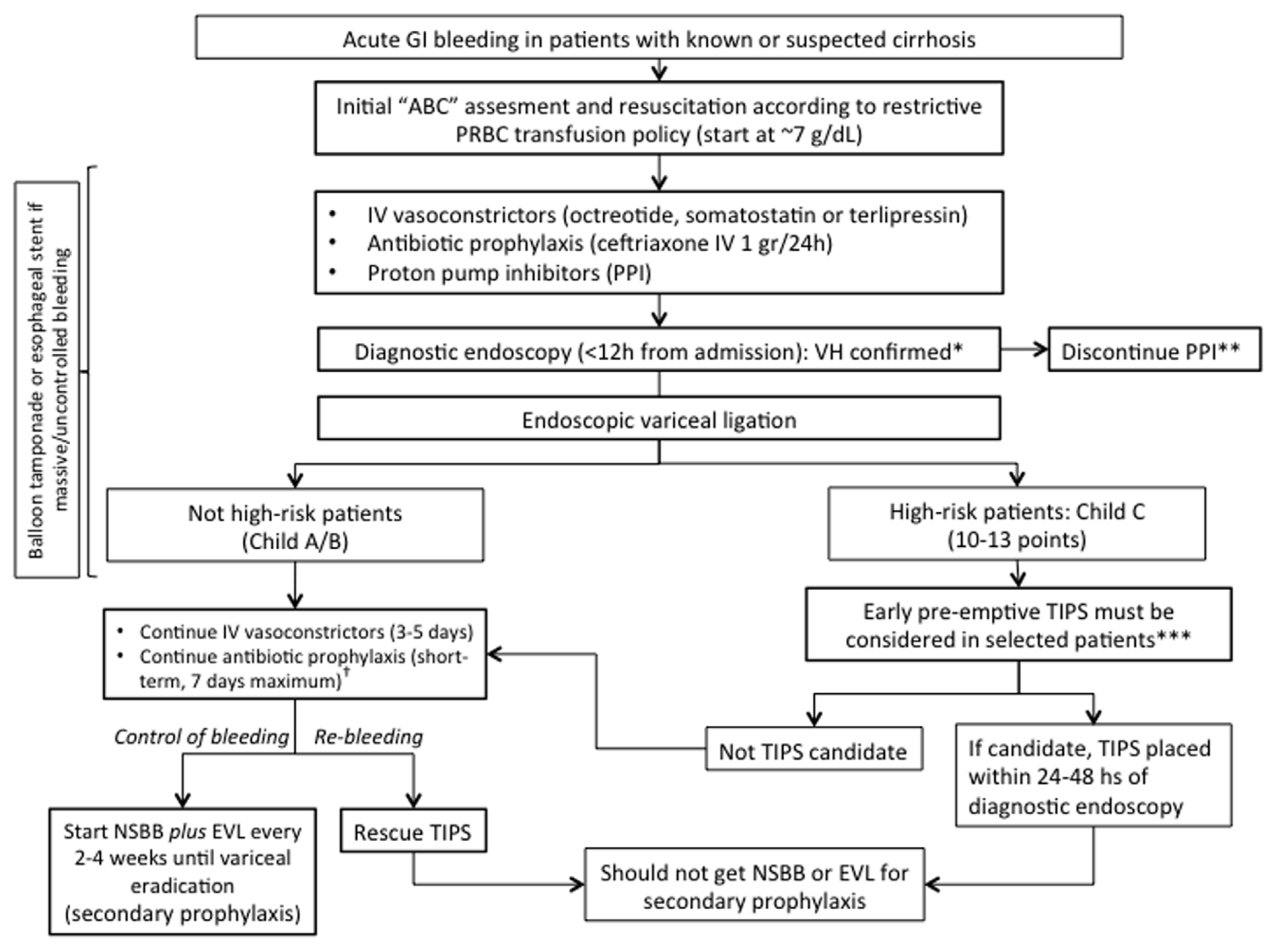
Algorithm for the management of acute gastrointestinal bleeding in patients with cirrhosis. ABC, airway, breathing, circulation; EVL, endoscopic variceal ligation; GI, gastrointestinal; IV, intravenous; NSBB, non-selective beta-blocker; PPI, proton pump inhibitor; PRBC, packed red blood cell; TIPS, transjugular intrahepatic portosystemic shunt; VH, variceal hemorrhage. *Any of the following: varix spurting blood, varices with overlying clot or with white nipple sign, varices and no other lesion that would explain hemorrhage. **A short-term course (10 days) of PPI may reduce the size of post-banding ulcers. ***Excluding patients who are more than 75 years old or who have hepatocellular carcinoma outside Milan criteria, creatinine level of at least 3 mg/dL, previous combination pharmacological plus endoscopic treatment to prevent re-bleeding, bleeding from isolated gastric or ectopic varices, recurrent hepatic encephalopathy, pulmonary hypertension, or heart failure or a combination of these. †Patient should not be discharged on prophylactic antibiotic (consider discontinuing at same time as vasoactive drugs).

**TIPS in patients at high risk of failing standard therapy (pre-emptive TIPS)** Patients with acute VH who are more likely to fail standard therapy are those with an HVPG of more than 20 mm Hg ^35^ or those belonging to Child C class ^32, 33^ or both. Therefore, it was postulated that use of TIPS *before* failure of standard therapy (so-called “pre-emptive TIPS”) would reduce mortality in these patients. A first small RCT in which 52 patients with HVPG of more than 20 mm Hg were randomly assigned to standard treatment versus pre-emptive TIPS (uncovered) ^36^ demonstrated significantly lower failure rates and short-term mortality in the TIPS arm. Later on, when covered stents became the standard of care for TIPS, a second multicenter RCT confirmed an improvement in survival in the TIPS group. Importantly, in this study ^37^, high-risk patients were defined as those in the Child C class with a score of 10 to 13 or those in the Child B class with active bleeding at the time of endoscopy. Several exclusion criteria were considered ( Figure 1), and only 20% of the patients initially evaluated were eligible for enrollment.

The favorable effect on survival of early TIPS has not been confirmed in observational cohorts ^38, 39^, and the survival benefit of pre-emptive TIPS was subsequently found to apply only to patients in the Child C group (score of 10 to 13) but not to those in the Child B class with active bleeding at endoscopy ^38^.

Therefore, candidates for pre-emptive TIPS appear to be those with Child C (score of 10 to 13). Within this group, those who are most likely to benefit need further elucidation. The feasibility of using model for end-stage liver disease (MELD) was evaluated in a retrospective cohort ^40^. Among the 206 patients who received early TIPS, those with MELD of at least 19 had a significant survival benefit.

Guidelines ^3, 7, 8^ recommend TIPS placement in the following patients at the time of acute VH:

1. Rescue TIPS in patients with persistent bleeding or early re-bleeding despite treatment with vasoconstrictors plus EVL.

2. Early (within 24 to 72 hours) pre-emptive TIPS can be considered in high-risk patients (Child C with score of less than 14) without contraindications to TIPS.

## Prevention of recurrent variceal hemorrhage

Patients who had a TIPS placed do not require further medical or endoscopic therapy for secondary prophylaxis but should be referred for evaluation of liver transplant in case they have other complications ^8^, and TIPS patency should be assessed at regular intervals by ultrasound together with screening for hepatocellular carcinoma.

Patients who recovered from an episode of esophageal VH can be divided in two distinct categories. The first includes patients who developed VH as the only decompensating event, at a relatively low risk of death. In this category, the aim of therapy is to prevent the occurrence of other complications (for example, ascites) in addition to preventing re-bleeding. The second category includes patients who had ascites or encephalopathy (or both) at the time of VH, at a very high risk of death. The goal of therapy in these patients is to improve survival. However, until now, RCTs in patients who had recovered from VH were not designed to account for this patient stratification or for these different outcomes. Hence, the following section is focused on the prevention of recurrent VH (“secondary prophylaxis”).

### First-line therapy

Combined therapy with non-selective beta-blockers (NSBBs) (propranolol or nadolol) plus EVL is the first-line therapy in the prevention of re-bleeding ^7, 8^, and NSBBs are the cornerstone of combined therapy. This recommendation is based on meta-analyses of RCTs performed to prevent re-bleeding. One such meta-analysis ^41^ showed that the added effect of NSBB to EVL improved the efficacy of EVL alone and reduced mortality but that the added effect of EVL to NSBB achieved only a non-significant decrease in re-bleeding without any effect on mortality. Later on, an individual meta-analysis ^42^ analyzed individual data from three trials comparing NSBB versus combination therapy and from four trials analyzing EVL versus combination therapy, thereby allowing patients to be stratified (Child A versus Child B/C). It showed that, in Child A patients (compensated), combination therapy was associated with lower all-source re-bleeding but without an effect on mortality. In Child B/C patients (mostly decompensated), however, combination therapy was associated with lower all-source re-bleeding rates but only in trials in which it was compared with EVL alone. This suggests that NSBB alone could be sufficient to prevent all-source re-bleeding in these patients. More importantly, mortality in trials in which combination therapy was compared with EVL alone was significantly lower, suggesting that NSBBs are essential in preventing not only re-bleeding but also death ^42^. The improvement in survival with NSBB is most likely related to a reduction in portal pressure that is associated not only with a reduction in the risk of variceal re-bleeding but also with a reduction in the development of other complications of portal hypertension, such as ascites and spontaneous bacterial peritonitis, as shown in a recent meta-analysis by Turco *et al*. ^43^.

Results from an individual patient data meta-analysis ^42^ demonstrate that NSBBs improve survival in patients with decompensated cirrhosis who have bled from varices. These data, obtained from RCTs, are in contrast to those of a cohort study that showed that, in patients with refractory ascites, mortality was higher in NSBB users than in non-users ^44^. However, in this cohort, groups were different at baseline (sicker in the NSBB group) and the determination of NSBB use was made at diagnosis of refractory ascites without information on NSBB use during follow-up. Many retrospective trials in different populations of patients with decompensated cirrhosis have been performed to confirm or refute these findings and two meta-analyses have summarized these data; both showed that NSBBs are not associated with a higher mortality ^45, 46^.

In studies that show a detrimental effect of NSBB ^44, 47^, the arterial pressure in NSBB users was significantly lower than in non-users and higher doses of propranolol had been used or a higher percentage of patients had been on carvedilol, suggesting that patients in whom there was clinical evidence of a negative inotropic effect or vasodilatory effect from NSBB/carvedilol were the ones who would be negatively affected ^48^. This would be expected as this clinically evident and probably dose-related deleterious hemodynamic effect would worsen the already-vasodilated state of patients with decompensated cirrhosis, particularly those with refractory ascites ^49^, leading to renal hypoperfusion, renal failure, and death.

Indeed, in a propensity-matched analysis of patients with refractory ascites, the use of propranolol was associated with increased survival, except for the subgroup on a high dose (at least 160 mg/day) ^50^.

Current guidelines recommend the combination of NSBB (propranolol or nadolol) plus EVL as first-line therapy to prevent recurrent VH, independent of the presence or absence of ascites/refractory ascites. Because of a lack of RCTs evaluating it for the prevention of variceal re-bleeding and because its additional vasodilating effect would be more deleterious in patients with decompensated cirrhosis who are more likely to be in the group requiring secondary prophylaxis, carvedilol is not recommended in this setting ^3, 8^. Propranolol and nadolol should be used cautiously in patients with ascites and should be started at a lower dose than in patients without ascites, and the maximum dose should be capped at a lower dose: propranolol should be capped to 160 mg/day (320 mg/day in patients without ascites) and nadolol to 80 mg/day (160 mg/day in patients without ascites) ^8^. Importantly, the dose of NSBB should be reduced or the drug should be discontinued in patients with refractory ascites who developed circulatory dysfunction defined by systolic blood pressure of less than 90 mm Hg, serum sodium of less than 130 mEq/L, or acute kidney injury ^8^.

### Second-line therapy

In patients who re-bleed despite being on secondary prophylaxis with NSBB plus EVL, TIPS is the treatment of choice ^3, 7, 8^. Uncovered ^51– 53^ and covered ^54, 55^ TIPS have been compared with NSBBs plus EVL as first-line treatment for secondary prophylaxis. Although this treatment is very effective in preventing re-bleeding, studies have shown a higher risk of encephalopathy and no differences in survival. The addition of simvastatin to standard of care was associated with a significant improvement in survival in Child A and B patients, although no difference of re-bleeding rate was observed ^56^. However, safety concerns were raised because patients with severe liver dysfunction had a higher-than-expected incidence of rhabdomyolysis. Unless future studies confirm these results, simvastatin cannot be recommended.

Patients who experience the first episode of VH while on primary prophylaxis with NSBBs have a higher risk of rebleeding and mortality compared to those who experience VH not on NSBB, despite being treated with recommended combination therapy ^57^. Even though the best approach in these patients is not known, they might benefit from an aggressive strategy, and TIPS may be considered instead of NSBB/EVL.

In patients with cirrhosis and PVT who have recently bled, variceal obliteration with EVL takes longer and varices recur at a higher rate compared with patients without PVT ^33, 58, 59^. Additionally, a small RCT ^60^ shows that TIPS is more effective than EVL plus NSBB in the prevention of re-bleeding in patients with cirrhosis and PVT with a higher likelihood of thrombus resolution but without differences in survival. Therefore, TIPS should be considered earlier in patients with PVT after VH, particularly in those awaiting liver transplantation, in whom the presence of PVT has been associated with a high risk of post-transplant mortality ^61^. Current guidelines recommend TIPS (covered) as the therapy of choice in those who experience re-bleeding despite combination therapy with NSBB plus EVL ^3, 7, 8^.

## Closing remarks

The management of varices and acute VH must be taken in the context of the severity of portal hypertension and the presence (or absence) of other complications related to cirrhosis or portal hypertension or both. Over the last decades, the advances in the therapy of portal hypertension have resulted in lower rates of decompensation and death, particularly for therapies associated with a decrease of portal pressure. In the future, risk stratification and improvements in therapies of patients with cirrhosis and acute VH are expected.

## Abbreviations

EVL, endoscopic variceal ligation; GI, gastrointestinal; Hb, hemoglobin; HVPG, hepatic venous pressure gradient; IV, intravenous; MELD, model for end-stage liver disease; NSBB, non-selective beta-blocker; PPI, proton pump inhibitor; PT, prothrombin time; PVT, portal vein thrombosis; RCT, randomized control trial; TIPS, transjugular intrahepatic portosystemic shunt; VH, variceal hemorrhage
